# Back to the Future of Soil Metagenomics

**DOI:** 10.3389/fmicb.2016.00073

**Published:** 2016-02-10

**Authors:** Joseph Nesme, Wafa Achouak, Spiros N. Agathos, Mark Bailey, Petr Baldrian, Dominique Brunel, Åsa Frostegård, Thierry Heulin, Janet K. Jansson, Edouard Jurkevitch, Kristiina L. Kruus, George A. Kowalchuk, Antonio Lagares, Hilary M. Lappin-Scott, Philippe Lemanceau, Denis Le Paslier, Ines Mandic-Mulec, J. Colin Murrell, David D. Myrold, Renaud Nalin, Paolo Nannipieri, Josh D. Neufeld, Fergal O'Gara, John J. Parnell, Alfred Pühler, Victor Pylro, Juan L. Ramos, Luiz F. W. Roesch, Michael Schloter, Christa Schleper, Alexander Sczyrba, Angela Sessitsch, Sara Sjöling, Jan Sørensen, Søren J. Sørensen, Christoph C. Tebbe, Edward Topp, George Tsiamis, Jan Dirk van Elsas, Geertje van Keulen, Franco Widmer, Michael Wagner, Tong Zhang, Xiaojun Zhang, Liping Zhao, Yong-Guan Zhu, Timothy M. Vogel, Pascal Simonet

**Affiliations:** ^1^Environmental Microbial Genomics Group, Laboratoire Ampère, Centre National de la Recherche Scientifique, UMR5005, Institut National de la Recherche Agronomique, USC1407, Ecole Centrale de Lyon, Université de LyonEcully, France; ^2^Research Unit for Environmental Genomics, Helmholtz Zentrum München Deutsches Forschungszentrum für Gesundheit und Umwelt (GmbH)Neuherberg, Germany; ^3^Aix-Marseille Université, CEA, Centre National de la Recherche Scientifique, Laboratoire d'Écologie Microbienne de la Rhizosphère et Environnements Extrêmes, UMR 7265, Biologie Végétale et de Microbiologie EnvironnementalesSaint-Paul-lez-Durance, France; ^4^Earth and Life Institute, Catholic University of LouvainLouvain-la-Neuve, Belgium; ^5^School of Life Sciences and Biotechnology, Yachay Tech UniversityUrcuquí, Ecuador; ^6^Natural Environment Research Council, Centre for Ecology and HydrologyOxford, UK; ^7^Laboratory of Environmental Microbiology, Institute of Microbiology of the Czech Academy of SciencesPraha, Czech Republic; ^8^Institut National de la Recherche Agronomique, US1279, Etude du Polymorphisme des Génomes Végétaux, CEA, Institut de Génomique, Centre National de GénotypageEvry, France; ^9^NMBU Nitrogen Group, Department of Chemistry, Biotechnology and Food Science, Norwegian University of Life SciencesAas, Norway; ^10^Earth and Biological Sciences Directorate, Pacific Northwest National LaboratoryRichland, WA, USA; ^11^Department of Plant Pathology and Microbiology, The Faculty of Agriculture, Food and Environment, The Otto Warburg-Minerva Center in Agricultural Biotechnology, The Hebrew University of JerusalemRehovot, Israel; ^12^Enzymology of Renewable Biomass, VTT, Technical Research Centre of FinlandEspoo, Finland; ^13^Ecology and Biodiversity, Institute of Environmental Biology, Utrecht UniversityUtrecht, Netherlands; ^14^Departamento de Ciencia Biológicas, Facultad de Ciencias Exactas, Instituto de Biotecnología y Biología Molecular, Centro Científico Tecnológico-Consejo Nacional de Investigaciones Científicas y Técnicas, Universidad Nacional de La PlataLa Plata, Argentina; ^15^Department of Biosciences, Swansea UniversitySwansea, UK; ^16^Institut National de la Recherche Agronomique, UMR 1347, Agroécologie, Université de BourgogneDijon, France; ^17^CEA/Direction des sciences du vivant/Institut de Génomique. Genoscope, Centre National de la Recherche Scientifiue UMR 8030, Université d'Evry Val d'EssonneEvry, France; ^18^Department of Food Science and Technology, Biotechnical Faculty- University of LjubljanaLjubljana, Slovenia; ^19^School of Environmental Sciences, University of East AngliaNorwich, UK; ^20^Department of Crop and Soil Science, Oregon State UniversityCorvallis, OR, USA; ^21^NALINOVDremil Lafage, France; ^22^Department of Agrifood and Environmental Science, University of FlorenceFlorence, Italy; ^23^Department of Biology, University of WaterlooWaterloo, ON, Canada; ^24^BIOMERIT Research Centre, School of Microbiology, National University of IrelandCork, Ireland; ^25^School of Biomedical Science, Curtin UniversityPerth, WA, Australia; ^26^National Ecological Observatory NetworkBoulder, CO, USA; ^27^Center for Biotechnology, Institute for Genome Research and Systems Biology, Genome Research of Industrial Microorganisms, Bielefeld UniversityBielefeld, Germany; ^28^Genomics and Computational Biology Group, René Rachou Research Centre – CPqRR/FIOCRUZBelo Horizonte, Brazil; ^29^Department of Environmental Protection, Estación Experimental del Zaidín, Consejo Superior de Investigaciones CientíficasGranada, Spain; ^30^Federal University of PampaSão Gabriel, Brazil; ^31^Archaea Biology and Ecogenomics Division, Department of Ecogenomics and Systems Biology, University of ViennaVienna, Austria; ^32^Center for Biotechnology and Faculty of Technology, Computational Metagenomics, Bielefeld UniversityBielefeld, Germany; ^33^Health and Environment Department, Bioresources, AIT Austrian Institute of Technology GmbHTulln, Austria; ^34^School of Natural Sciences and Environmental Studies, Södertörn UniversityHuddinge, Sweden; ^35^Section of Genetics and Microbiology, Department of Plant and Environmental Microbiology, University of CopenhagenFrederiksberg, Denmark; ^36^Section of Microbiology, Department of Biology, University of CopenhagenCopenhagen, Denmark; ^37^Thünen-Institute of BiodiversityBraunschweig, Germany; ^38^Agriculture and Agri-Food Canada, Department of Biology, University of Western OntarioLondon, ON, Canada; ^39^Department of Environmental and Natural Resources Management, University of PatrasAgrinio, Greece; ^40^Department of Microbial Ecology, Groningen Institute for Evolutionary Life Sciences, University of GroningenGroningen, Netherlands; ^41^Institute of Life Science, Medical School, Swansea UniversitySwansea, UK; ^42^Molecular Ecology, Institute for Sustainability Sciences, AgroscopeZürich, Switzerland; ^43^Department of Microbiology and Ecosystem Science, Division of Microbial Ecology, University of ViennaVienna, Austria; ^44^Environmental Biotechnology Laboratory, Department of Civil Engineering, The University of Hong KongHong Kong, China; ^45^Group of Microbial Ecology and Ecogenomics, State Key Laboratory of Microbial Metabolism, School of Life Sciences and Biotechnology, Shanghai Jiao Tong UniversityShanghai, China; ^46^Institute of Urban Environment, Chinese Academy of SciencesXiamen, China

**Keywords:** metagenomic, soil microbiology, terrestrial microbiology, soil ecology, microbial ecology

Direct extraction and characterization of microbial community DNA through PCR amplicon surveys and metagenomics has revolutionized the study of environmental microbiology and microbial ecology. In particular, metagenomic analysis of nucleic acids provides direct access to the genomes of the “uncultivated majority.” Accelerated by advances in sequencing technology, microbiologists have discovered more novel phyla, classes, genera, and genes from microorganisms in the first decade and a half of the twenty-first century than since these “many very little living animalcules” were first discovered by van Leeuwenhoek (Table 1). The unsurpassed diversity of soils promises continued exploration of a range of industrial, agricultural, and environmental functions. The ability to explore soil microbial communities with increasing capacity offers the highest promise for answering many outstanding who, what, where, when, why, and with whom questions such as: Which microorganisms are linked to which soil habitats? How do microbial abundances change with changing edaphic conditions? How do microbial assemblages interact and influence one another synergistically or antagonistically? What is the full extent of soil microbial diversity, both functionally and phylogenetically? What are the dynamics of microbial communities in space and time? How sensitive are microbial communities to a changing climate? What is the role of horizontal gene transfer in the stability of microbial communities? Do highly diverse microbial communities confer resistance and resilience in soils?

**Table 1:** **Timeline of advances in genomic and metagenomic methods and large-scale projects focusing on soil biodiversity analysis: cracking the soil black box**.

**Date**	**Advances**	**References**
1980	Direct extraction and purification of DNA from soil opening the world of soil molecular ecology	Torsvik, 1980
1990	DNA re-association experiments revealing the magnitude of genetic diversity in soil to be above 4000 different genomes per cm^3^	Torsvik et al., 1990
1992	First description of fluorescent *in situ* hybridization (FISH) method using rRNA sequence as a taxon specific probe applied to a soil environment	Hahn et al., 1992
1998	Description of a new method for cloning high-molecular weight soil DNA in bacteria artificial chromosome for bioactive molecules mining and first use of the term “metagenomic”	Handelsman et al., 1998
2005	First soil DNA cloning and shotgun sequencing study generating 100 Mbp of data	Tringe et al., 2005
2006	The first soil metatranscriptomic study using cDNA high-throughput sequencing to investigate active ammonia oxidizers	Leininger et al., 2006
2007	Metatranscriptomic investigation of soil poly-adenylated cDNA revealing eukaryotic microbes functional diversity	Bailly et al., 2007
2009	Announcement of the TerraGenome consortium	Vogel et al., 2009
2009	High-throughput genetic screening of a soil fosmid library by probe hybridization on high-density membranes	Demanèche et al., 2009
2010	Announcement of the Earth Microbiome Project	Gilbert et al., 2010
2014	Assembly attempt of one of the biggest soil sequencing efforts to date illustrating the major computational challenges associated with large and complex sequence datasets	Howe et al., 2014
2014	Announcement of the Brazilian Microbiome Project	Pylro et al., 2014
2014	Announcement of the China Soil Microbiome Initiative	(http://english.issas.cas.cn/)
2015	Assembly of nearly complete genomes from a prairie soil using a microcosm enrichment approach	Delmont et al., 2015
2015	Alaska permafrost soil study combining targeted 16S rRNA gene, metagenomic and metatranscriptomics sequencing as well as shotgun mass-spectrometry analysis of metaproteomics	Hultman et al., 2015

Although molecular techniques, including metagenomics, have revolutionized the study of microbial ecology, the sheer magnitude of soil microbial diversity has prevented full access to the scope and scale of relevant microbiology questions worth asking of this complex habitat. Indeed, we still lack the ability to link most microorganisms to their metabolic roles within a soil community. Increased sequencing capacity provided by high-throughput sequencing technologies has helped characterize and quantify soil diversity, yet these methodologies are commonly leveraged to process additional samples at a relatively shallow depth rather than survey all genomes from a single sample comprehensively. In addition to high diversity, methodological biases remain an enormous challenge for microbial community characterization. These biases include soil sampling, DNA extraction, adsorption of nucleic acids to soil particles, contributions of extracellular DNA, sample preparation, sequencing protocols, sequence analysis, and functional annotation. Because current sequencing technologies generate millions of reads with each analysis, hurdles associated with interpreting these “big data” can add to the challenges faced by microbial ecologists in understanding soils and the involvement of different microorganisms in the range of services that soils provide.

Microbial surveys, such as the Earth Microbiome Project (EMP; Gilbert et al., 2014), TerraGenome (Vogel et al., 2009), the Brazilian Microbiome Project (Pylro et al., 2014), the China Soil Microbiome Initiative (http://english.issas.cas.cn/), EcoFINDERS (http://ecofinders.dmu.dk/), and MicroBlitz (http://www.microblitz.com.au/) are good examples of large-scale coordinated efforts to explore soil taxonomic and functional diversity (Table 1). Nonetheless, the degree to which data from these consortia reflect original soil sample community compositions is unknown. Illustrating the extent of this problem, soil DNA extraction methods are described in over 100 articles, yet no single criterion (e.g., quantity of DNA, quality of DNA, composition of DNA, sequence diversity) can be used as a benchmark for extraction and recovery efficiency because no single “true” reference or benchmark for soil microbial community composition has been validated to date.

Without a suitable benchmark methodology or dataset for confirming the fidelity of amplicon or metagenomic analyses, assessing whether the presence and activity of organisms are correctly evaluated is impossible. In this way, metagenomic exploration of soil microbial diversity is analogous to satellite remote sensing of Earth's biodiversity with defective satellites. Consider a hypothetical survey of African savannah biodiversity by a satellite that cannot detect mammals, leading the observer to overlook a herd of water buffalo in a watering hole that was also colonized by a flock of pink flamingos; even browsed grass and compacted soil might simply be attributed to flamingos. In contrast, another flamingo-replete watering hole might have very tall grass and healthy soil. Thus, this one narrow view would prevent the accurate survey-based establishment of cause and effect (i.e., water buffaloes graze grass and compact soil). The satellites and their results are akin to soil DNA extraction techniques and sequence data, respectively. Furthermore, methodological limitations that may prevent the detection of some abundant and active bacteria in soil might lead to the same critical level of misinterpretation caused by a biased satellite overlooking the buffaloes responsible for soil compaction. While an observer in the savannah would immediately infer the state of the soil is due to the buffaloes, soil microbiologists cannot benefit from the in situ observer insight and might associate (erroneously) the unseen “buffalo” activity to any observed “flamingo” bacteria. This means that the use of limited techniques (flawed satellites and DNA extraction protocols) could have severe consequences on both the underestimation of microbial biodiversity and our understanding of the functional role of unobserved key players including associating critical activities to the wrong organisms. The use of alternate soil treatment protocols is like using other satellites with potentially different flaws, including an inability to detect birds, insects, or snakes. Each DNA extraction technique has its own bias that might produce additional apparent relationships. No single protocol/satellite would be considered sufficient in isolation. Therefore, the discovery of ecological principles would be strengthened when supported by sequence data/satellite imagery from multiple time points and multiple satellites. Even though comparing different ecosystems with the same satellite would be unlikely to identify the relationship between the presence of water buffalo and grazed grass, or soil compaction, all data collected from all satellites would increase the probability that a more representative list of animal biodiversity could be generated. Similarly, the taxonomic and potentially functional deciphering of the soil microbiota would critically benefit from a combination of methods.

Although conservation biologists can circumvent satellite data and benchmark remote observations by direct watering hole and savannah investigations, the single cell genomics approach requires significant technical development to physically isolate and sequence every microorganism in soil; the other meta–omics approaches (transcriptomics, proteomics, metabolomics) are also strongly affected by biases. In addition, identifying water buffalos, pink flamingos, and most other animals is considerably easier than the enormously Sisyphean task of interpreting metagenomic sequence data, measuring microbial diversity, and assigning putative functions to recovered metagenomes or small subunit (SSU) rRNA gene sequences. These challenges are exacerbated by the availability of only a few thousand bacterial genomes in public databases for comparison, akin to distinguishing a thousand distinct buffalo species that all look the same from satellite imagery alone. With differences in soil chemistry, plant cover, and underlying bedrock geology, there is no simple way to identify relative differences in soil DNA extraction efficiency from one sample vs. another. The relative distribution of microbial populations deduced from a soil DNA extract may overestimate rare populations and extracellular DNA at the expense of abundant but lysis-recalcitrant bacteria. Microbiologists may well be missing 99% of soil microbial populations in exchange for capturing microbial “flamingos” that are far more readily detected.

Using amplicon surveys or metagenomic approaches for comparing soil microbial communities and correlating indicator species with specific environmental perturbations or specific land usage tends to produce statistically valid trends whether the selection of the different methods minimize the bias of subsequent results or not. However, different DNA extraction techniques, amplification methodologies, sequencing protocols, bioinformatic analyses, databases used for comparing and annotating sequences—all of these steps influence both the qualitative and quantitative results of molecular surveys and metagenomics (Delmont et al., 2013). True replicates cannot be performed because of soil compositional changes, even at the micro-scale level; one gram of soil is not the same as another. Another challenge is that the total number of species present in a single sample of soil is completely unknown, with wildly variable estimates. Even identifying all species present (i.e., “alpha diversity”) has not been accomplished for any single soil sample; no soil microbial “species” accumulation curve has yet reached an asymptote. The first question of the five “Ws” (i.e., who is where?) remains unanswered for soil microbiologists.

Soil microbiologists are faced with substantial challenges, a little bit like the hero of the famous 1985 movie “Back to the future” who, after having been accidently sent back to the past, must adapt his actions to make the future possible. There is no silver bullet for soil metagenomics, but there are possible experimental approaches that could help quantify the extent of methodological bias, define ecological theories, and provide a more solid foundation for future studies.

One important first step toward addressing some of the issues faced by soil microbiologists is to begin generating a comprehensive catalog of all microbial community members and functions for at least one reference soil. Such a relatively complete reference dataset would shed light on the as-yet-unknown shape of a soil microbial species frequency distribution and could serve as a future reference for assessing community composition changes across soil landscapes (i.e., beta diversity). In other words, the extent of bias with any individual approach (i.e., a single DNA extraction method) could be explicitly determined by comparing extraction methods coupled with comprehensive characterization of the selected reference soil. The objectives should include identifying minimally biased methods (or combinations of methods) for soil characterization, differentiating between active soil microorganisms and dormant cells (and extracellular DNA), assessing seasonal variability, and quantifying the full scope and scale of soil microbial taxonomic and functional diversity, including the diversity of “rare biosphere” microorganisms that typically dominate assessments of soil microbial diversity (Lynch and Neufeld, 2015).

The reverse engineering of a reference soil could also generate additional discoveries through complementary datasets. For example, including the isolation and characterization of cells via single-cell genomics can help target phylogenetically distinct microbial “dark matter” from this reference soil, as has been demonstrated recently for selected aquatic samples (Rinke et al., 2013). Experimental and computational techniques (Albertsen et al., 2013; Howe et al., 2014) for the assembly of complete genomes by differential abundance binning of metagenomic data could be enabled by large datasets derived from multiple extraction methods. Coupled with comprehensive DNA-based characterization of the collected reference soil microbial community, this research initiative should ideally also assess multiple levels of gene expression, at the level of RNA (metatranscriptomics), proteins (metaproteomics), and metabolites (metametabolomics). Together, these complementary datasets would converge toward an exhaustive inventory of all microbial taxa and functional genes present in a single soil or several reference soils, offering powerful insight into soil taxonomic and functional structure at a scale thought impossible even a decade ago. By identifying how a reference soil community is structured, both spatially and temporally, the information from this coordinated effort could help provide missing links between conventional soil analyses and the underlying composition of soil microbial communities.

In-depth exploration of a single reference soil must involve experiments far beyond the usual metagenomic analyses applied to soil samples. Instead, this initiative will require extensive benchmarking of the sampling strategy itself, which is linked to identifying a suitable reference site and exploring the spatial heterogeneity of the selected soil microbial community. Several soil systems are ideal candidates for acting as a reference soil, including the internationally recognized agroecology field site in Rothamsted, UK (Torsvik, 1980; Vogel et al., 2009; Delmont et al., 2012) and one of the American native prairie soils investigated by high throughput sequencing (Fierer et al., 2013; Howe et al., 2014). The number and size of the samples must be carefully adapted at different spatial (gram, core, field, landscape) and temporal (seasonal variation) scales in conjunction with experimental constraints related to sieving and homogenization of the largest samples, without neglecting the local soil heterogeneity down to the smallest microstructures. Such an endeavor would require a coordinated interdisciplinary consortium of expertise spanning microbiology, biochemistry, soil physics and chemistry, genomics, metagenomics, bioinformatics, and molecular biology. The results of the initiative could form an objective basis for establishing standardized protocols for future and ongoing soil microbiological investigations. Indeed, we argue that this reductionist reverse engineering approach to soil microbiology and broad scale surveys are synergistic and that these approaches should be performed in parallel. In doing so, fundamental knowledge gathered on the reference soil would serve to aid future soil survey efforts, reducing bias and increasing objectivity for analysis and comparison of multiple samples.

The scientific community requires both reductionist approaches and broad scale surveys to better describe soil microbial communities, understand microbial dynamics, explore microbial and environmental interrelationships, detect and decipher microbial diversity, discover functions that can be exploited for industry and agriculture, and elucidate microbial adaptation and evolution within the context of soil services. Microbial ecologists have been dependent on the interpretation of limited data, akin to microbial satellite imagery, for far too long. The extent of methodological bias remains unknown and a comprehensive catalog of soil microorganisms and functional genes does not yet exist for any soil. We still do not know the extent of what we do not know. There are more than a million times as many soil microorganisms on our planet than stars in the universe and we argue that the time has come for humans to tackle the challenge of soil microbial diversity.

## Author contributions

JN, TV, and PS proposed the manuscript's idea. JN, TV, PS, WA, SA, MB, PB, DB, AF, TH, JJ, EJ, GK, KK, AL, HL, DL, PL, IM, JM, DM, RN, PN, JDN, FO, JP, AP, VP, JR, LR, CS, MS, AS, AS, SS, JS, SJS, CT, ET, GT, JV, GV, MW, FW, TZ, XZ, LZ, and YZ wrote the manuscript and acknowledged the final version.

## Funding

JN was funded by a fellowship from the French MENESR.

### Conflict of interest statement

The authors declare that the research was conducted in the absence of any commercial or financial relationships that could be construed as a potential conflict of interest.
